# Thyme and Savory Essential Oil Vapor Treatments Control Brown Rot and Improve the Storage Quality of Peaches and Nectarines, but Could Favor Gray Mold

**DOI:** 10.3390/foods7010007

**Published:** 2018-01-05

**Authors:** Karin Santoro, Marco Maghenzani, Valentina Chiabrando, Pietro Bosio, Maria Lodovica Gullino, Davide Spadaro, Giovanna Giacalone

**Affiliations:** 1 Department of Agricultural, Forestry and Food Sciences (DISAFA), University of Turin, Largo Paolo Braccini 2 (ex-Via L. da Vinci 44), 10095 Grugliasco, Italy; karin.santoro@unito.it (K.S.); marco.maghenzani@unito.it (M.M.); valentina.chiabrando@unito.it (V.C.); marialodovica.gullino@unito.it (M.L.G.); giovanna.giacalone@unito.it (G.G.); 2 AGROINNOVA—Centre of Competence for the Innovation in the Agro-environmental Sector, University of Turin, Largo Paolo Braccini 2 (ex-Via L. da Vinci 44), 10095 Grugliasco, Italy; pietro.bosio@unito.it

**Keywords:** biofumigation, *Monilinia* spp., *Botrytis* spp., essential oils, stone fruit, postharvest disease

## Abstract

The effect of biofumigation, through slow-release diffusors, of thyme and savory essential oils (EO), was evaluated on the control of postharvest diseases and quality of peaches and nectarines. EO fumigation was effective in controlling postharvest rots. Naturally contaminated peaches and nectarines were exposed to EO vapors for 28 days at 0 °C in sealed storage cabinets and then exposed at 20 °C for five days during shelf-life in normal atmosphere, simulating retail conditions. Under low disease pressure, most treatments significantly reduced fruit rot incidence during shelf-life, while, under high disease pressure, only vapors of thyme essential oil at the highest concentration tested (10% *v*/*v* in the diffusor) significantly reduced the rots. The application of thyme or savory EO favored a reduction of brown rot incidence, caused by *Monilinia fructicola*, but increased gray mold, caused by *Botrytis cinerea*. In vitro tests confirmed that *M. fructicola* was more sensitive to EO vapors than *B. cinerea*. Essential oil volatile components were characterized in storage cabinets during postharvest. The antifungal components of the essential oils increased during storage, but they were a low fraction of the volatile organic compounds in storage chambers. EO vapors did not influence the overall quality of the fruit, but showed a positive effect in reducing weight loss and in maintaining ascorbic acid and carotenoid content. The application of thyme and savory essential oil vapors represents a promising tool for reducing postharvest losses and preserving the quality of peaches and nectarines.

## 1. Introduction

Peaches and nectarines (*Prunus persica* (L.) Batsch) are fruit rich in vitamins, fibers and other phytochemical compounds, such as carotenes and polyphenols, which are important for a healthy diet [1,2]. Stone fruit are consumed worldwide and represent one of the most important fruit. In recent years, peach and nectarine production increased progressively [3] and the main global producer is China with over 12 million tons, followed by Spain and Italy, which produce 3 million tons annually [4]. Peaches and nectarines are the 3rd most important fruit crop in the European Union after apples and pears [5]. During storage, the quality characteristics and some commercial parameters of these fruit can decrease, at a rate depending on the storage conditions, due to postharvest diseases and senescence processes [6]. 

The most common postharvest pathogens on stone fruit are *Monilinia* spp. (*M. fructigena* Honey, *M. fructicola* (G. Winter) Honey, and *M. laxa* (Aderh. and Ruhland) Honey), agents of brown rot, *Botrytis cinerea* Pers. agent of gray mold, *Penicillium expansum* Link, agent of blue mold, *Alternaria* spp., agent of black mold, and *Rhizopus stolonifer*, Ehrenb., agent of Rhizopus rot [7,8]. On peaches, postharvest losses can cause even higher damage than preharvest diseases [9]. Crop protection in Europe should be performed in the orchard because only one fungicide (fludioxonil) can be used on stone fruit after harvesting, but the supermarket chains typically request either no further postharvest treatments or a limited number of active ingredients, as residues, on the fruit. The last fungicide has to be applied 1 or 2 weeks before harvesting, to guarantee a high level of fruit protection against pathogens during storage, and to remain below the maximum residue limits imposed by European legislation [10]. Moreover, consumer attention is attracted by environmentally friendly production practices, preferring foods treated with natural products instead of conventional pesticides [11]. At present, *Monilinia* spp. control depends on an integrated strategy based on orchard fungicide spray programs and maintenance of proper storage conditions in the packinghouse and during distribution [12].

Hence, intense research efforts focus on developing innovative, unconventional, sustainable strategies to preserve fruit quality and decrease food losses. The most promising control approaches are the use of microorganisms and natural products with intrinsic antimicrobial properties [13,14]. Essential oils (EOs) represent a powerful tool to reduce the environmental impact of fruit production [15]. The efficacy of plant EOs has been extensively evaluated in vitro [16], but a few studies have been performed in vivo [17]. However, these treatments may modify the organoleptic characteristics of fruit, changing taste or flavor during cold storage.

The antifungal activity of EOs is determined by their chemical composition. In particular, aldehydes, phenols and ketones considerably inhibit pathogen growth. Thymol, carvacrol and *p*-anisaldehyde have a proven fungicidal activity and EOs rich in these components showed the highest inhibitory activity against many postharvest pathogens, such as *Penicillium digitatum* [18], *Colletotrichum gloeosporioides* [19] and *R. stolonifer* [20]. EOs of thyme (*Thymus vulgaris*) and savory (*Satureja montana*) are mainly composed of thymol and carvacrol [21], which are highly effective in controlling fungal pathogens. Generally, the efficacy of EOs is investigated through direct contact with fruit, by application through spraying or dipping [22]. However, these application methods can have undesired effects, including phytotoxicity and organoleptic modification of treated fruit. Only a few studies have reported the efficacy of EO treatments through biofumigation, focusing on phytopathological aspects [23]. Scientific research recently started to pay particular attention to the assessment of the antifungal activity of EO vapors [24,25]. Fumigation with thyme and savory EOs resulted in effective control of several postharvest pathogens, showing antifungal activity against *Colletotrichum* [26], *Aspergillus*, *Penicillium*, *Mucor* and *Trichoderma* spp. [27] The use of EOs through fumigation avoids the direct contact with the product, reducing the possibility of influencing the sensorial profile.

The aim of this study was to investigate the effect of the vapor phase of thyme and savory EOs, applied by fumigation through slow-release diffusors, on both quality parameters and postharvest diseases of peaches and nectarines, during cold storage and shelf life. Savory and thyme solid diffusors were prepared at two different concentrations and they were used to treat naturally infected peaches and nectarines. In order to clarify how essential oils diffuse and persist in cold rooms, the atmospheric composition of the storage cabinets was analyzed during cold storage. At the same time, the antimicrobial activity of essential oil vapors was evaluated in vitro on conidial germination of *M. fructicola* and *B. cinerea*, two of the main postharvest pathogens of peaches and nectarines.

## 2. Materials and Methods

The EOs of thyme (*Thymus vulgaris*) and savory (*Satureja montana*) used in the assays were prepared by Soave (Turin, Italy). The compositional analyses were performed using a gas chromatograph Shimadzu GC-2010 Plus (Shimadzu, Kyoto, Japan) equipped with a mass spectrometer GCMS-QP 2010 Ultra (Shimadzu) and a split-splitless injector. The gas chromatograph was fitted with a 30 m × 0.25 mm fused silica capillary Zebron ZB-5MSi column (Phenomenex, Torrance, CA, USA) with 0.25 μm film thickness. Helium carrier gas using a linear velocity of 37 cm/s with a constant flow rate of 1.0 mL/min was used. The pressure was 55 kPa and total flow was 105 mL/min. Ion electron impact spectra at 70 eV were recorded in scan mode (30–700 m/z).

For savory essential oil, the oven program started with an initial temperature of 50 °C for 3 min, heating at 5 °C/min to 70 °C, 70 °C for 5 min, heating at 1 °C/min to 90 °C, heating at 5 °C/min to 150 °C, and finally heating at 40 °C/min to 270 °C, held for 5 min. For thyme essential oil, the same protocol was used without the isothermal phase at 70 °C for 5 min. For both essential oils, the injection temperature was set at 250 °C and the ion source and the interface were both set at 280 °C.

Pure essential oil of savory and thyme were diluted at 1% and 10% in *n*-hexane (VWR, Radnor, PA, USA) for direct injection using split mode (80%). Sampling in the chambers was performed using SPME fiber assembly 100 μm PDMS (Supelco Analytical, Bellefonte, PA, USA) for 5 min in triplicate at 1, 14 and 28 days of incubation whereby the injector was in splitless mode. Relative composition (percentage) of volatile compounds was calculated by comparing peak area to area of total chromatogram (from 7.5 to 40 min). Absolute quantification was calculated for carvacrol and thymolusing a standard calibration curve between 1 and 50 ppm (mg/L). Relative quantification was determined for the other compounds, by using standard calibration curve of thymol for thyme essential oil and standard calibration curve of carvacrol for savory essential oil.

Peaches (‘Vista Rich’) and nectarines (‘Sweet Red’) were harvested from two different orchards located in Lagnasco (Cuneo, Italy, 44°37′33″60 N, 07°33′21″24 E) at the firm-ripe stage and transported immediately to the laboratory of DISAFA, University of Torino, during the summer of 2015. All fruit were sorted by size. Defect-free fruit were randomly divided into five lots of 350 fruit each. Each lot was treated in a different way and further divided into 5 replicates of 70 fruit. Each replicate was a box kept in a container at the same temperature, but different atmosphere according to the treatment. 

EO diffusors were made by adding EO (10% *v*/*v*), sterilized deionized water (88% *v*/*v*) and Tween 20 (2% *v*/*v*) (Merck, Darmstadt, Germany) to agar-agar (Merck) (15 g/L). Lower EO concentrations were obtained by serial dilutions. 50 mL of medium were poured into Petri dishes and after agar solidification, 5 diffusors were installed in storage cabinets under the fruit boxes. Fruit were stored in refrigerator cabinets (75 × 70 × 65 cm) at 0 °C and 98% relative humidity for 28 days. Fumigation was performed at 1% and 10% EO concentrations. A total of four treatments were tested: thyme EO at 1%, thyme EO at 10%, savory EO at 1% and savory EO at 10%. An untreated control was included. The analyses were performed at 1, 14 and 28 days of cold storage. 

After harvesting, healthy sound fruit were selected. Rotten fruit were counted and incidence of diseased fruit was calculated for each treatment every 7 days up to 28 days of storage, and for 5 days of shelf life at 20 °C. Pathogens were isolated by transferring small pieces of symptomatic fruit tissues, previously washed in 1% sodium hypochlorite and rinsed in sterile deionized water, onto potato dextrose agar (PDA, Merck) plates amended with 25 mg/L streptomycin sulfate (Merck). A 7-day-old culture was used for DNA extraction by using the EZNA Plant DNA Kit (Omega Bio-Tek, Norcross, GA, USA). The internal transcribed spacer (ITS) region of rDNA of 50 isolates was amplified using the ITS1/ITS4 primers [28]. The PCR reaction mixture comprised 2 µL 10 × PCR buffer, 1 µL ITS1 primer at 10 mM, 1 µL of ITS4 primer at 10 mM, 1 µL of nucleotides mixture at 5 mM, 12 µL of MilliQ autoclaved water, 0.8 µL of MgCl_2_ at 25 mM, 0.2 µL of Taq polymerase and 2 µL of template DNA. PCR cycles included a denaturing step at 95 °C for 2 min and 35 cycles as follows: 94 °C for 30 s, 55 °C for 30 s, 72 °C for 1 min and a final elongation step at 72 °C for 7 min. ITS amplicons were sequenced by BMR Genomics (Padua, Italy), and DNA sequences were compared with those present in the NCBI database and deposited with accession numbers.

The effect of essential oils on conidial germination was investigated on two main peach and nectarine pathogens, *B. cinerea* and *M. fructicola*. Two virulent strains isolated from peaches were stored on agar slant at 4 °C until use. *B. cinerea* conidial suspension was obtained from 15 days of culture grown on PDA+ streptomycin 25 mg/L at 25 °C. *M. fructicola* was cultured on tomato agar plate (250 mL of tomato puree, 750 mL of deionized water and 20 g of agar) amended with 25 mg of streptomycin for 5 days. 5 mL of sterile deionized water were added to the plate and the mycelium was gently scraped with L-shaped spreader to detach the conidia. Conidial suspension was filtered through four layers of sterile cheesecloth and 100 µL were spread on PDA+ streptomycin plates. PDA plates were sealed with the essential oil diffusor. EOs were added to the diffusors at 350 µL/L and 35 µL/L, a concentration proportional to the quantity present in the cabinet considering the concentration of EO diffusor, the number of diffusors per cabinet and the volume of the cabinet and the plates. Conidial germination was assessed after 20 h for *B. cinerea* and 36 h for *M. fructicola* counting 100 conidia per plate. Three replicates were used for each treatment and the assay was repeated twice. Conidia were considered germinated when the germ tube exceeded the conidial length.

Weight loss was determined by weighing 30 fruit per treatment at the beginning of the trial (zero time) and during storage (7, 14, 21 and 28 days of storage, and at 5 days for shelf life). Values are reported as shown in Equation (1).

(1) $$\%\;weight\;loss=\frac{initial\;weight-final\;weight}{initial\;weight}\times 100$$

The color parameters were measured weekly during cold storage, with a Minolta chromameter (CR400, Konica Minolta Sensing, Inc., Osaka, Japan), calibrated with a standard white plate, using the CIE L*C*h (lightness, chroma/saturation and hue angle) scale. The surface of 30 fruit (ground and over color) was evaluated per treatment.

The carotenoid content was determined every 14 days, using the method reported by Talcott and Howard [29]. The carotenoids were extracted using 10 mL of extraction solvent (ethanol/acetone *w*/*w* with 0.2% butylhydroxytoluene) and 2 g of fresh sample from 10 fruit, homogenized at 24,000 rpm for 1 min using an Ultra-Turrax T-25 Tissue homogenizer (IKA^®^-Labortechnik, Saufen, Germany). The samples were then centrifuged at 2900 rpm for 20 min at 2 °C (Avanti J-25 centrifuge, Beckman Instruments, Palo Alto, CA, USA). The recovered supernatants were combined with 30 mL of extraction solvent, 25 mL of hexane and 12.5 mL of nanopure water. The tubes were left in the dark at 4 °C for 1 h, then the samples were transferred to quartz cuvettes. Absorbance was measured using a UV-visible spectrophotometer (DU 530, Beckman Coulter, Brea, CA, USA) at 450 nm, with β-carotene as an external standard. The results are expressed as μg β-carotene equivalents per kg weight of fruit and are an average of three replicates per treatment and per time point (at 0, 14 and 28 days of storage).

Ascorbic acid (AA) was determined according to Sánchez-Moreno et al. [30] and González-Molina et al. [31], and evaluated at 0, 14 and 28 days (three replicates per treatment and per time point). The ascorbic acid was extracted using 10 mL of extraction solvent (methanol:water 5:95 *v*/*v*) and 10 g of fruit flesh from 10 fruit per treatment by homogenization with a T-25 Ultra-Turrax for 3 min. Then, pH was adjusted to 2.2–2.4, and the extract was adsorbed onto a C18 Sep-Pak cartridge (Waters Associates, Milford, MA, USA). The resultant solution was added to 1,2-phenylenediamine dihydrochloride (Fluka Chemika, Neu-Ulm, Switzerland) and left to stand for 37 min before HPLC analysis. The AA and dehydroascorbic acid contents were expressed as mg/kg fresh weight. Three replicate analyses were performed on the flesh. The chromatographic system (Agilent Technologies, Inc., Santa Clara, CA, USA) was equipped a Kinetex-C18 column (4.6 × 150 mm, 5 μm, Phenomenex, Torrance, CA, USA), a pump and a diode array detector. The system was controlled through HPLC online software (Agilent Technologies, Inc.) at 40 °C. The mobile phase (isocratic) comprised 50 mM monobasic potassium phosphate and 5 mM cetrimide (Sigma-Aldrich Corporation, Saint Louis, MO, USA) in methanol:water (5:95 *v*/*v*). The flow rate was 0.9 mL/min. The detector was set at 261 nm for AA and 348 for dehydroascorbic acid (DHAA). External calibration curves for AA and DHAA, respectively, were used for quantification. The total run time was 15 min. The detection limit is 0.5 mg/kg of fresh weight.

Analysis of variance (ANOVA) was performed on the data using Statistica v. 6.0 (Statsoft Inc., Tulsa, OK, USA). Least significant differences (LSD) at a significance level of 0.05 were used to compare treatment means with Tukey’s test. Mean values for each time point were considered significantly different at *p* ≤ 0.05. 

## 3. Results and Discussion

The vapor phase activity makes EOs potential biofumigants of fruit in storage chambers. Slow release diffusors, based on EO gel emulsions, were used to release EO vapor phase during the storage of peaches and nectarines. Pathogens were not inoculated, so only natural pathogen inoculum could develop both on nectarines and peaches. 

**Figure 1:**
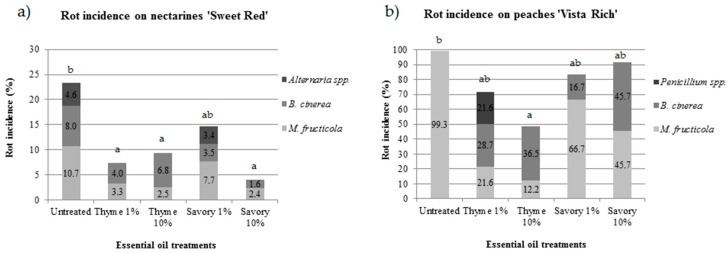
Rot incidence in nectarines ‘Sweet Red’ (**a**) and peaches ‘Vista Rich’ (**b**) treated with essential oil biofumigation and pathogen incidence (%) at the end of shelf life (5 days at 20 °C after 28 days of cold storage). Values of the same storage trial, followed by the same letter, are not statistically different by Tukey’s Test (*p* < 0.05).

EO fumigation was effective in controlling postharvest rots of peaches and nectarines, but the efficacy was different in the two experimental conditions. During cold storage, no rots developed either on nectarines ‘Sweet Red’ or on ‘Vista Rich’ peaches. Rot incidence on nectarines ‘Sweet Red’ and peaches ‘Vista Rich’ greatly differed during shelf life (5 days at 20 °C). After shelf life, on nectarines the rot incidence in untreated fruit was 23.3%, while most peaches (99.3%) were rotten in untreated control (Figure 1).

Disease incidence was influenced by the weather conditions during fruit growing. A high incidence of brown rot on peaches, which were harvested one month before nectarines, was favored by fresh and humid weather conditions, while nectarines were harvested in August, after a period characterized by high temperatures and scarce precipitations, which limited the development of brown rot [32].

Untreated control showed the highest percentage of rotten fruit in both trials. The pathogens isolated from rotten fruit were identified using morphological and molecular tools. On nectarines, the postharvest pathogens isolated from untreated fruit were *Monilinia fructicola*, *Botrytis cinerea*, and *Alternaria* spp. (Table 1), with an incidence of 10.7%, 8.0% and 4.6%, respectively.

The efficacy of the EO treatments was influenced by the disease incidence: under low disease pressure, three out of four treatments significantly reduced the fruit rot incidence, while under high disease pressure, only vapors of thyme at 10% significantly reduced the rots compared to the control.

On nectarines, savory EO at 10% was the most effective treatment, with only 4.0% of the fruit damaged by postharvest pathogens. All the treatments were effective in controlling postharvest diseases, except for savory EO at 1%, which was not statistically different from the control at the end of the shelf life (Figure 1a). Thyme EO was very effective in controlling *M. fructicola*. The increase in EO concentration resulted in a decrease of brown rot. *B. cinerea* was isolated with an incidence of 4.0% and 6.8% for 1% and 10% thyme EO treatment, respectively. 

On nectarines, *M. fructicola* was the major pathogen in untreated fruit (47.2% of rotten fruit), while the relative incidence decreased to 45.0% and 26.8% in fruit treated with thyme EO at 1% and 10%, respectively. *B. cinerea* affected 34.3%, 55.0% and 73.2% of rotten fruit in the control, in thyme at 1% and at 10%, respectively. After the first report of *M. fructicola* in Italy in 2009 [33], the pathogen has mainly substituted the endemic species *M. laxa* in Italian orchards. 

Moreover, *Alternaria* spp. was not isolated in fruit fumigated with thyme EO. Savory EO vapors were less effective in controlling this pathogen because only 10% concentration inhibited *Alternaria* spp. growth, while 3.4% of fruit fumigated with 1% savory EO showed *Alternaria* rot. Reduced activity against *Alternaria* spp. was reported for savory essential oil, moreover the essential oil at 400 ppm favored *Alternaria* rot [34]. *Alternaria* spp. is a secondary pathogen of nectarines and the presence on the samples is mainly due to long storage.

On peaches, under high disease pressure, untreated fruit were completely damaged by brown rot. On fruit fumigated with EOs, *M. fructicola*, *B. cinerea* and *Penicillium* spp. were isolated (Figure 1b). The efficacy of thyme EO against *M. fructicola* depended on its concentration. *M. fructicola* affected only 30% and 25% of the rotten fruit fumigated with 1% and 10% thyme EO, respectively. 

*B. cinerea*, the causal agent of gray mold, is uncommon on peaches, but its presence can be higher when the incidence of brown rot is low [35]. The application of thyme or savory EO, both on nectarines and on peaches favored a reduction of brown rot incidence, but a concomitant increase of gray mold. 

In accordance with the literature, *M. fructicola* was very sensitive to thyme essential oil [36]. The fungicidal properties of thyme EO are mainly due to thymol, recognized as the most active phenolic compound against postharvest molds [37].

The chemical composition of the EO used in the trial was determined by direct injection. Principal volatile compounds present in pure thyme essential oil were thymol (26.0%), followed by *p*-cymene, α-terpineol and linalool (Table 2).

As diffusors, based on EO gel emulsions, were used to release EO vapor phase during the fruit storage, we wanted to determine the concentration of EO compounds in the atmosphere of the chambers during fruit cold storage. The analyses performed in chambers with thyme EO diffusors at 1% and 10% showed a very high amount of *p*-cymene, the main volatile organic compound at 1, 14 and 28 days. Thymol, instead, was on average 2.0% during the three samplings in thyme EO at 10%, while in thyme EO at 1% thymol was 0.40%, 3.66% and 3.67% at day 1, 14 and 28, respectively. Thymol in storage chambers was released slowly and at lower concentration, compared to the pure essential oil composition. This experiment demonstrated that thymol was slowly released by diffusors during the 28 days of fruit storage in cold chambers, and persisted by increasing in absolute concentration during storage. 

Regarding savory essential oil, direct injection showed that the principal volatile compound was linalool (22.2%), followed by carvacrol (13.3%), thymol (10.7%) and eucalyptol (Table 3).

In storage room atmosphere, where savory diffusors at 10% were used, linalool and eucalyptol were the main components at the three sampling times. On the contrary, thymol and carvacrol in chambers showed a significant decrease compared to the pure EO, in fact they were present at 0.14% to 0.63% for carvacrol and at 0.17% to 0.60% for thymol at the beginning and at the end of the trial, respectively. 

These results showed that antifungal components of thyme and savory essential oils increased during storage, but they were a low fraction of the volatile organic compounds in the storage chambers, compared to their concentration in pure EO. For this reason, diffusors could be considered effective in a slow release of vapor EOs.

**Figure 2:**
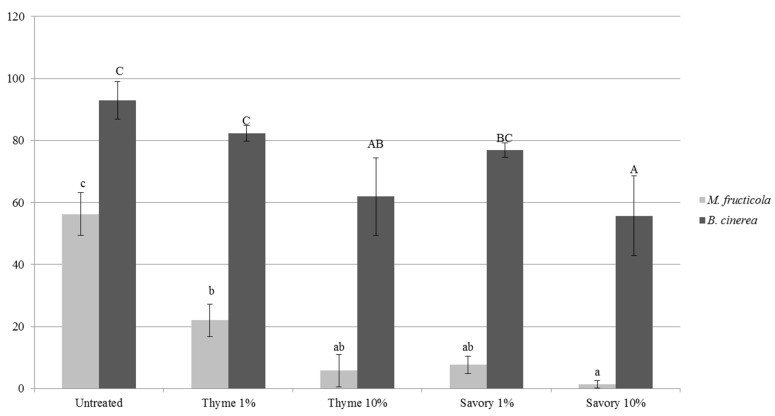
*M. fructicola* (gray columns) and *B. cinerea* conidial germination percentage (black columns) after biofumigation without direct contact with essential oil in vitro. Conidial germination was assessed after 20 h for *B. cinerea* and 36 h for *M. fructicola* at 20 °C. Values of the same pathogen, followed by the same letter, are not statistically different by Tukey’s Test (*p* < 0.05).

The antifungal activity of thyme and savory EO in vapor phase was demonstrated by performing an in vitro test in agar plate, where *M. fructicola* and *B. cinerea* could germinate in an atmosphere with the same concentration present in the cabinet (Figure 2). 

The antifungal effects depended on the type and concentration of EO, and on the pathogen species [17]. *M. fructicola* was almost completely inhibited by savory vapors at 10%. Thyme essential oil vapors led to 40% decrease of conidial germination rate. As expected, vapor treatments were more effective in vitro, when EO concentration increased. All the EO treatments caused a significant decrease of germ tube length with respect to the untreated control (Table 4). 

Previous results showed that thymol vapors inhibited *M. fructicola* mycelial growth and were fatal for conidia viability; the fumigated spores resulted shrunken and with collapsed protoplasts [38]. Thymol is able to crystallize on the outer surface of cell walls and exposed fungal structures were characterized by disrupted cell membranes and disorganized cytoplasmic organelles, reducing to 50% conidial viability at 2 µg/mL and to 17–23% when thymol was applied at 8 µg/mL [39].

*B. cinerea* is much less sensitive to essential oil antimicrobial activity [40]. In our experiments, the fungus confirmed to be less sensitive to EO vapors, compared to *M. fructicola* and maximum reduction of conidial germination was 40.1% for savory vapors at 10%. Germ tube length of *B. cinerea* growth was inhibited only by 10% thyme treatment, while the other treatments were not statistically different from the untreated control. 

In vitro tests confirmed the results obtained in fruit: the antimicrobial activity of vapor EOs does not need a direct contact. Moreover, the higher efficacy against *M. fructicola*, compared to *B. cinerea* in vitro explained the higher reduction of brown rot on fruit, accompanied by a higher incidence of gray mold. This effect was particularly evident on peaches. Untreated fruit were affected exclusively by *M. fructicola,* while other pathogens could develop on fumigated fruits by colonizing the niche left empty by the inhibition of *M. fructicola*. Thanks to the lower sensitivity of *B. cinerea* to EO vapors, the pathogen could start to develop on fumigated fruit causing gray rots. 

Besides the efficacy against the postharvest rots, weight loss, color, total soluble solids, titratable acidity, ascorbic acid and carotenoids contents were analyzed during fruit storage.

The use of thyme EO reduced weight loss particularly on peaches (Table 5).

Untreated control showed the highest weight loss at the end of the storage in both trials. The efficacy of EO fumigation could be related to the formation of a coating on the fruit surface that modifies gas permeation, reducing respiration rate and water loss. Our results were in agreement with previous experiments on cherries [41], grapes [42], and peaches [43] treated with eugenol, thymol, menthol and cinnamon vapors. The EO vapors were shown to reduce the dehydration process or weight loss in fruit [44]. Other studies showed that the application of essential oils together with edible coatings could reduce the water loss [45]. There are few references reporting the effect of EO treatments on fruit color. Here, ground and overcolor lightness and hue angle were monitored during storage. The lightness values of the nectarines decreased both in ground and overcolor during cold storage (Table 6). 

The differences among the treatments were observed at the end of the shelf life, when treated samples showed higher values than the untreated control. Regarding peaches, no differences among the treatments were detected (Table 7). 

The hue angle is a measure of ripening. In general, the hue angle values of the ground color decrease during postharvest storage, as fruit become yellow. In nectarines, the hue angle remained stable during storage, but some differences among treatments were observed and nectarines treated with EOs were yellower than the control fruit. In peaches, the ground color became yellower during storage as expected, but no differences among treatments were observed. In conclusion, the use of EOs had no negative effect on fruit color. The color variations were connected with the natural ripening process and were slowed down by cold storage. 

**Figure 3:**
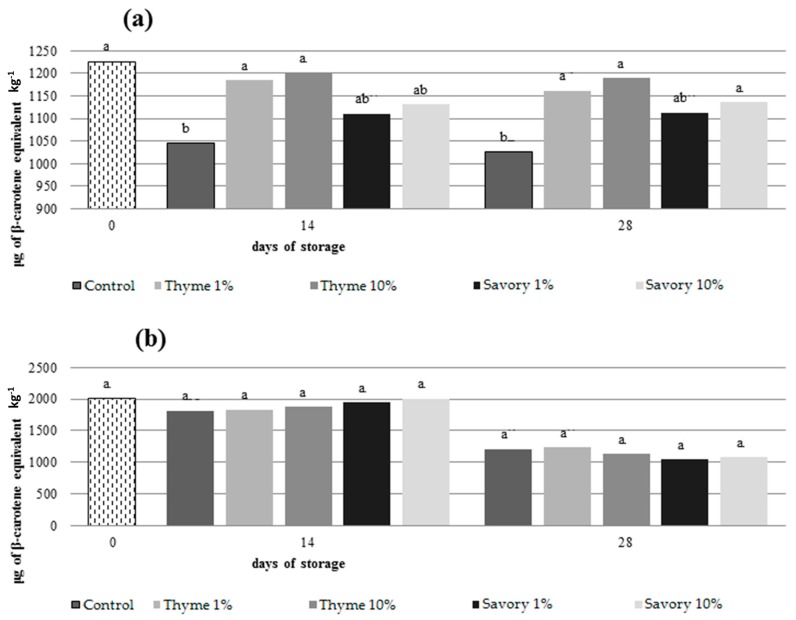
Total content of carotenoids in nectarines ‘Sweet Red’ (**a**) and peaches ‘Vista Rich’ (**b**) after 0, 14 and 28 days of cold storage. Mean values at the same time followed by the same letter are not significantly different by Tukey’s Test at *p* ≤ 0.05.

EO vapors influenced also carotenoid content preserving the amount of these antioxidant compounds in fumigated fruit. Generally, carotenoids decrease during storage. They are photo- and heat-sensitive and tend to oxidize if they are not protected from light and atmosphere [46]. In nectarines, only untreated control and fruit treated with savory EO at 1% showed a significant decrease in total carotenoid content during storage, while carotenoid amounts remained stable in the other fumigated fruit (Figure 3). 

At the end of the trials, fruit treated with thyme at 1% and 10% had the highest amount of carotenoids (1189 μg/kg and 1162 μg/kg, respectively). 

**Figure 4:**
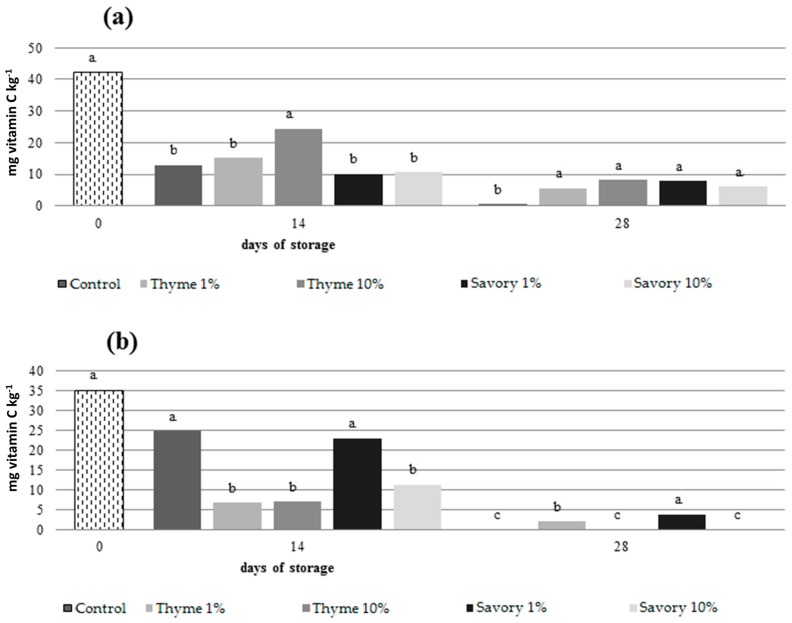
Total content of vitamin C (ascorbic acid and dehydroascorbic acid) in nectarines ‘Sweet Red’ (**a**) and peaches ‘Vista Rich’ (**b**) after 0, 14 and 28 days of cold storage. Mean values at the same time followed by the same letter are not significantly different by Tukey’s Test at *p* ≤ 0.05.

Both on nectarines and peaches, untreated fruit showed the lowest AA content at the end of cold storage resulting statistically different from treated fruit (Figure 4).

The use of EOs preserved ascorbic acid contents, without showing statistical differences between the treatments. In peaches, fruit fumigated with savory and thyme essential oils at 1% maintained the grater ascorbic acid content at the end of the trial. 

The ascorbic acid content decreased significantly during cold storage, as expected, and the EO influenced the process. The decrease in ascorbic acid could be explained by the chemical properties and metabolic functions of AA in the plant cell [47]. AA and dehydroascorbic acid (DHAA) are important antioxidants and are used against the reactive oxygen species, produced naturally by the metabolism of vegetal cells, and accumulated during storage [48]. 

The use of EOs led to a delay in the loss of total carotenoids content and AA in the pulp. Some EOs (thymol, menthol and eugenol) have shown antioxidant activity and free radical scavenging capability [49]. These effects could explain the EO efficacy in reducing the loss of nutrients. Valero et al. (2006) [50] suggested that the addition of thymol slowed the AA loss in grape. Nevertheless, different effects of EOs on total carotenoids content between nectarines and peaches were evident. The EOs were more effective on the metabolism of nectarines, while in peaches the action of the EOs was less evident.

## 4. Conclusions

Savory and thyme essential oil vapors demonstrated a high antifungal activity against *M. fructicola* even at low concentrations. *B. cinerea* was less sensitive to EO vapors and could develop when *M. fructicola* was inhibited. EO concentrations require a careful optimization depending on fruit cultivars. Although in in vitro tests the efficacy is directly correlated with EO concentrations, when applied on fruit EO vapors could have phytotoxic effects, which reduce treatment efficacy. EO vapors did not influence the overall quality of the fruit, but showed a positive effect in maintaining nutritional values avoiding ascorbic acid and carotenoids oxidation, preserving the quality of the final product. The application of thyme and savory essential oils represents a promising tool for reducing postharvest losses and preserving quality in peaches and nectarines.

## Figures and Tables

**Table 1:** GenBank accession numbers of ITS sequences of some pathogenic isolates from nectarines ‘Sweet Red’.

Isolated Strains	GenBank Accession Number
CaF1—*Botrytis cinerea*	KX304007
S1cF3—*Botrytis cinerea*	KX304008
CcF5—*Monilinia fructicola*	KX304009
S1aF1—*Monilinia fructicola*	KX304010
Cb22—*Alternaria* sp.	KX304011
Cc12—*Alternaria* sp.	KX304012

**Table 2:** Thyme essential oil composition and volatilized compounds in storage cabinet at 1, 14 and 28 days.

EO * Components	DI ** (%)	Thyme 10%						Thyme 1%					
		T1		T14		T28		T1		T14		T28	
		ppm	(%)	ppm	(%)	ppm	(%)	ppm	(%)	ppm	(%)	ppm	(%)
α-Pinene	3.59	17.35	2.59	35.32	4.19	48.44	4.64	3.76	4.07	8.31	2.10	12.74	2.60
Camphene	0.98	7.27	1.05	15.76	1.84	21.53	2.04	1.25	1.06	2.40	0.54	4.71	0.91
β-Pinene	0.30	3.29	0.45	6.53	0.74	8.58	0.79	0.36	0.00	1.49	0.30	2.24	0.39
Myrcene	0.89	12.17	1.80	18.66	2.19	29.39	2.80	2.18	2.18	4.25	1.03	7.05	1.40
*p*-Cimene	23.52	321.89	49.07	372.43	44.56	397.13	38.30	53.21	63.38	78.13	20.59	99.92	20.89
Linalool	8.00	96.47	14.67	135.54	16.19	166.85	16.07	3.17	3.36	94.47	24.91	100.66	21.04
Fenchol	0.45	5.55	0.79	9.23	1.06	13.69	1.29	0.36	0.00	5.39	1.33	6.00	1.18
Terpinen-1-ol	2.30	14.71	2.19	22.45	2.65	31.92	3.05	0.87	0.61	19.67	5.11	24.85	5.14
Camphor	0.37	3.01	0.40	4.07	0.44	6.96	0.64	1.88	1.82	3.31	0.78	4.07	0.78
β-Terpineol	1.03	6.58	0.95	11.02	1.28	15.79	1.49	0.60	0.28	9.20	2.34	11.39	2.31
Isoborneol	0.71	9.05	1.33	13.79	1.61	19.85	1.88	0.36	0.00	9.77	2.49	11.43	2.32
Borneol	1.90	12.65	1.88	22.71	2.68	29.65	2.83	0.36	0.00	29.14	7.62	22.55	4.65
Terpinen-4-ol	0.67	5.36	0.76	7.32	0.83	10.79	1.01	0.36	0.00	6.00	1.49	7.01	1.39
α-Terpineol	13.40	30.12	4.54	53.57	6.37	74.71	7.18	1.51	1.37	50.54	13.28	64.49	13.45
γ-Terpineol	2.60	6.73	0.97	10.74	1.24	14.32	1.35	0.36	0.00	11.19	2.86	13.30	2.71
Thymol	26.02	4.28	0.60	23.56	2.78	28.07	2.67	0.69	0.40	14.17	3.66	17.87	3.67

* EO: essential oil; ** DI: disease index.

**Table 3:** Savory essential oil composition and volatilized compounds in storage cabinet at 1, 14 and 28 days.

EO * Components	DI ** (%)	Savory 10%						Savory 1%					
		T1		T14		T28		T1		T14		T28	
		ppm	(%)	ppm	(%)	ppm	(%)	ppm	(%)	ppm	(%)	ppm	(%)
α-Pinene	0.76	4.64	0.48	16.27	0.76	12.06	0.64	1.35	0.20	2.03	0.23	3.63	0.40
Myrcene	0.47	5.33	0.56	15.19	0.70	16.70	0.89	1.31	0.19	1.59	0.16	5.15	0.59
*p*-Cymene	10.63	119.89	13.71	297.20	14.17	248.17	13.62	34.46	7.43	41.12	5.78	55.77	6.96
Eucalyptol	7.71	185.98	21.30	442.63	21.11	394.67	21.67	138.52	30.15	113.11	16.01	160.06	20.09
γ-Terpinen	9.45	128.33	14.68	284.79	13.57	234.37	12.86	38.00	8.20	46.39	6.53	62.31	7.79
*trans*-Linalool oxide	1.19	11.95	1.32	42.58	2.01	51.48	2.81	2.15	0.38	9.50	1.29	12.76	1.55
*cis*-Linalool oxide	0.86	9.49	1.04	29.57	1.39	37.81	2.05	1.63	0.26	6.31	0.83	9.31	1.12
Linalool	22.16	183.71	21.03	418.00	19.93	384.31	21.10	138.19	30.08	286.32	40.63	273.29	34.33
Plinol	0.34	4.47	0.46	12.46	0.57	13.33	0.71	2.90	0.54	6.90	0.92	7.25	0.86
Isoborneol	1.09	10.89	1.20	29.90	1.41	31.05	1.68	7.20	1.48	18.72	2.60	18.43	2.26
Borneol	2.24	13.44	1.49	32.25	1.52	36.66	1.99	9.48	1.98	23.08	3.22	27.94	3.46
α-Terpineol	1.36	4.51	0.47	10.31	0.47	12.11	0.64	3.64	0.70	7.27	0.97	9.71	1.17
Carvone	2.09	7.26	0.78	15.48	0.72	17.10	0.92	8.24	1.70	16.99	2.35	16.76	2.05
Bornyl acetate	1.55	9.84	1.08	20.52	0.96	16.98	0.91	11.08	2.32	12.70	1.74	10.59	1.28
Thymol	10.67	1.59	0.17	6.65	0.36	9.43	0.60	1.65	0.34	4.78	0.75	8.77	1.27
Carvacrol	13.29	1.68	0.14	10.38	0.47	11.90	0.63	2.19	0.38	6.41	0.85	11.66	1.41

* EO: essential oil; ** DI: disease index.

**Table 4:** Essential oil vapors effect on germ tube length in vitro measured after 36 h at 20 °C for *M. fructicola* and 20 h at 20 °C for *B. cinerea*.

	*M. fructicola* Germ Tube (µm)	*B. cinerea* Germ Tube (µm)
Untreated control	10.5 ^b^	212.7 ^c^
Thyme 1%	8.0 ^a^	208.5 ^bc^
Thyme 10%	6.5 ^a^	190.9 ^ab^
Savory 1%	8.6 ^a^	165.6 ^ab^
Savory 10%	5.8 ^a^	109.9 ^a^

Values of the same column, followed by the same letter within each column, are not statistically different by Tukey’s Test (*p* < 0.05).

**Table 5:** Weight loss (% *w*/*w*) of nectarines ‘Sweet Red’ and peaches ‘Vista Rich’.

Fruit	Treatments	Weight Loss (% *w*/*w*)				
		Days of Storage				
		7	14	21	28	5 Shelf Life
Nectarines	Untreated control	0.55 ^ab^	0.82 ^a^	1.06 ^a^	1.29 ^a^	10.7 ^a^
Nectarines	Thyme 1%	0.80 ^a^	1.00 ^a^	1.20 ^a^	1.35 ^a^	9.01 ^b^
Nectarines	Thyme 10%	0.69 ^ab^	0.85 ^a^	1.04 ^a^	1.20 ^a^	9.22 ^ab^
Nectarines	Savory 1%	0.55 ^ab^	0.79 ^a^	1.00 ^a^	1.15 ^a^	9.46 ^ab^
Nectarines	Savory 10%	0.49 ^b^	0.72 ^b^	0.94 ^a^	1.16 ^a^	9.49 ^ab^
Peaches	Untreated control	1.55 ^a^	1.91 ^a^	2.16 ^a^	2.51 ^a^	-
Peaches	Thyme 1%	1.31 ^a^	1.42 ^c^	1.72 ^c^	1.81 ^c^	-
Peaches	Thyme 10%	0.99 ^b^	1.26 ^d^	1.49 ^d^	1.61 ^c^	-
Peaches	Savory 1%	1.39 ^a^	1.73 ^b^	1.97 ^b^	2.10 ^b^	-
Peaches	Savory 10%	1.54 ^a^	1.77 ^ab^	1.97 ^b^	2.10 ^b^	-

Mean values followed by the same letter within each column are not significantly different by Tukey’s Test at *p* ≤ 0.05.

**Table 6:** Color parameters (lightness and hue angle) in nectarines with different treatments during storage.

Color Parameters	Ground or Overcolor	Treatments	Storage Times (Days)					
			0	7	14	21	28	5 Shelf Life
Lightness (L*)	GC	Untreated control	68.70 ^a^	67.86 ^a^	67.78 ^a^	68.6 ^a^	68.28 ^a^	63.71 ^b^
	GC	Thyme 1%	67.75 ^a^	65.38 ^a^	65.64 ^a^	67.57 ^a^	65.97 ^ab^	64.96 ^ab^
	GC	Thyme 10%	67.67 ^a^	65.32 ^a^	65.37 ^a^	67.17 ^a^	65.64 ^b^	64.07 ^ab^
	GC	Savory 1%	68.79 ^a^	65.55 ^a^	66.81 ^a^	67.18 ^a^	67.67 ^ab^	67.75 ^a^
	GC	Savory 10%	68.38 ^a^	66.85 ^a^	65.79 ^a^	67.84 ^a^	68.14 ^ab^	66.78 ^ab^
Hue angle (h)	GC	Untreated control	82.44 ^a^	86.25 ^a^	83.73 ^a^	84.12 ^a^	84.25 ^a^	66.16 ^a^
	GC	Thyme 1%	80.28 ^a^	79.73 ^b^	78.39 ^a^	77.91 ^b^	78.98 ^ab^	66.50 ^a^
	GC	Thyme 10%	78.76 ^a^	75.11 ^b^	79.01 ^a^	77.74 ^b^	78.03 ^b^	69.73 ^a^
	GC	Savory 1%	81.59 ^a^	79.55 ^b^	79.37 ^a^	81.31 ^ab^	81.82 ^ab^	70.29 ^a^
	GC	Savory 10%	83.24 ^a^	81.47 ^ab^	81.43 ^a^	81.35 ^ab^	77.94 ^b^	72.89 ^a^
Lightness (L*)	OC	Untreated control	40.20 ^a^	48.11 ^a^	41.64 ^a^	38.34 ^b^	39.79 ^a^	44.19 ^ab^
	OC	Thyme 1%	44.73 ^a^	46.96 ^a^	39.00 ^ab^	42.03 ^a^	40.12 ^a^	39.63 ^b^
	OC	Thyme 10%	40.16 ^a^	42.41 ^b^	37.45 ^b^	42.65 ^a^	37.45 ^a^	43.93 ^ab^
	OC	Savory 1%	45.20 ^a^	43.14 ^b^	37.54 ^b^	35.68 ^b^	38.76 ^a^	48.14 ^a^
	OC	Savory 10%	39.34 ^a^	45.96 ^ab^	37.81 ^ab^	37.54 ^b^	36.73 ^a^	43.72 ^ab^
Hue angle (h)	OC	Untreated control	20.28 ^a^	42.58 ^a^	33.75 ^a^	27.32 ^b^	30.88 ^a^	29.58 ^ab^
	OC	Thyme 1%	33.94 ^a^	41.84 ^ab^	32.78 ^a^	34.52 ^a^	31.80 ^a^	20.80 ^c^
	OC	Thyme 10%	25.68 ^a^	36.69 ^b^	31.23 ^a^	29.67 ^b^	28.32 ^a^	28.27 ^bc^
	OC	Savory 1%	33.49 ^a^	38.3 ^ab^	30.79 ^a^	27.46 ^b^	29.16 ^a^	36.20 ^a^
	OC	Savory 10%	25.19 ^a^	40.64 ^ab^	33.05 ^a^	28.48 ^b^	27.07 ^a^	27.47 ^bc^

Nectarines ‘Sweet Red’: GC: ground color; OC: over color. Mean values followed by the same letter within each column are not significantly different by Tukey’s Test at *p* ≤ 0.05. Letters in the same column are used to compare the treatment influence.

**Table 7:** Color parameters (lightness and hue angle) in peaches with different treatments during storage.

Color Parameters	Ground or Overcolor	Treatments	Storage Times (Days)				
			0	7	14	21	28
Lightness (L*)	GC	Untreated control	49.16 ^a^	49.30 ^a^	49.42 ^b^	54.45 ^a^	53.44 ^a^
	GC	Thyme 1%	49.42 ^a^	50.50 ^a^	51.72 ^ab^	54.24 ^a^	52.97 ^a^
	GC	Thyme 10%	49.43 ^a^	51.55 ^a^	51.52 ^ab^	52.96 ^a^	53.61 ^a^
	GC	Savory 1%	49.62 ^a^	52.08 ^a^	54.46 ^ab^	55.38 ^a^	55.20 ^a^
	GC	Savory 10%	49.20 ^a^	51.07 ^a^	54.46 ^a^	54.04 ^a^	52.85 ^a^
Hue angle (h)	GC	Untreated control	43.12 ^a^	57.21 ^a^	60.76 ^b^	68.78 ^a^	65.62 ^a^
	GC	Thyme 1%	46.06 ^a^	63.87 ^a^	67.13 ^ab^	70.48 ^a^	64.16 ^a^
	GC	Thyme 10%	52.56 ^a^	60.00 ^a^	68.88 ^ab^	66.61 ^a^	66.40 ^a^
	GC	Savory 1%	52.84 ^a^	65.96 ^a^	71.44 ^a^	75.48 ^a^	71.30 ^a^
	GC	Savory 10%	48.81 ^a^	59.94 ^a^	69.11 ^ab^	69.25 ^a^	62.33 ^a^
Lightness (L*)	OC	Untreated control	37.98 ^a^	41.38 ^c^	43.23 ^a^	45.56 ^a^	45.08 ^a^
	OC	Thyme 1%	36.56 ^a^	44.20 ^ab^	44.28 ^a^	43.57 ^a^	45.39 ^a^
	OC	Thyme 10%	34.24 ^a^	44.79 ^ab^	43.09 ^a^	45.12 ^a^	44.14 ^a^
	OC	Savory 1%	34.86 ^a^	45.30 ^a^	42.15 ^a^	43.93 ^a^	45.73 ^a^
	OC	Savory 10%	33.79 ^a^	42.77 ^bc^	43.54 ^a^	43.74 ^a^	46.35 ^a^
Hue angle (h)	OC	Untreated control	15.22 ^a^	35.06 ^bc^	36.60 ^a^	36.52 ^a^	34.26 ^a^
	OC	Thyme 1%	18.45 ^a^	39.76 ^a^	36.98 ^a^	35.14 ^a^	37.52 ^a^
	OC	Thyme 10%	17.25 ^a^	37.91 ^ab^	35.01 ^a^	37.18 ^a^	34.56 ^a^
	OC	Savory 1%	22.42 ^a^	38.01 ^ab^	33.45 ^a^	35.07 ^a^	36.34 ^a^
	OC	Savory 10%	17.88 ^a^	32.04 ^c^	35.02 ^a^	34.98 ^a^	35.70 ^a^

Peaches ‘Vista Rich’. GC: ground color; OC: over color. Mean values followed by the same letter within each column are not significantly different by Tukey’s Test at *p* ≤ 0.05. Lowercase letters (a, b) in the same column are used to compare the treatment influence.
